# Ursodeoxycholic and chenodeoxycholic bile acids alleviate endotoxininduced acute lung injury in rats by modulating aquaporin expression and pathways associated with apoptosis and inflammation

**DOI:** 10.3389/fphar.2025.1484292

**Published:** 2025-03-06

**Authors:** Tatjana Milivojac, Milkica Grabež, Ljiljana Amidžić, Alma Prtina, Aleksandra Krivokuća, Ugljesa Malicevic, Maja Barudžija, Milka Matičić, Snežana Uletilović, Nebojša Mandić-Kovačević, Tanja Cvjetković, Miloš P. Stojiljković, Milica Gajić Bojić, Momir Mikov, Radoslav Gajanin, Sergey Bolevich, Aleksandar Petrović, Ranko Škrbić

**Affiliations:** ^1^ Centre for Biomedical Research, Faculty of Medicine, University of Banja Luka, Banja Luka, Bosnia and Herzegovina; ^2^ Department of Hygiene, Faculty of Medicine, University of Banja Luka, Banja Luka, Bosnia and Herzegovina; ^3^ Department of Pathophysiology, Faculty of Medicine, University of Banja Luka, Banja Luka, Bosnia and Herzegovina; ^4^ Department of Histology and Embryology, Faculty of Medicine, University of Banja Luka, Banja Luka, Bosnia and Herzegovina; ^5^ Department of Medical Biochemistry and Chemistry, Faculty of Medicine, University of Banja Luka, Banja Luka, Bosnia and Herzegovina; ^6^ Department of Pharmacy, Faculty of Medicine, University of Banja Luka, Banja Luka, Bosnia and Herzegovina; ^7^ Department of Pharmacology, Toxicology and Clinical Pharmacology, Faculty of Medicine, University of Banja Luka, Banja Luka, Bosnia and Herzegovina; ^8^ Department of Pharmacology, Toxicology and Clinical Pharmacology, Faculty of Medicine, University of Novi Sad, Novi Sad, Serbia; ^9^ Department of Pathologic Physiology, I.M. Sechenov First Moscow State Medical University, Moscow, Russia; ^10^ Department of Histology and Embryology, Faculty of Medicine, University of Nis, Nis, Serbia

**Keywords:** chenodeoxycholic acid, ursodeoxycholic acid, endotoxin, acute lung injury, oxidative stress, inflammation, apoptosis, aquaporins

## Abstract

**Introduction:**

This study aimed to investigate the anti-inflammatory, antioxidant, and anti-apoptotic properties of ursodeoxycholic (UDCA) and chenodeoxycholic (CDCA) bile acids in a rat model of endotoxin (lipopolysaccharide, LPS)-induced acute lung injury (ALI).

**Methods:**

The study included six groups of Wistar rats exposed to different pretreatments. The control and endotoxin groups were pretreated with propylene glycol, a solvent for bile acids, while the other groups received UDCA or CDCA for 10 days. On the 10th day, an endotoxin injection was given to evaluate the impact of these pretreatments. Lung tissue sections were analyzed by immunohistochemistry, targeting the pro-inflammatory marker nuclear factor kappa B (NF-κB), the anti-apoptotic marker B-cell lymphoma 2 (BCL-2), pro-apoptotic markers BCL-2-associated X protein (BAX) and caspase 3, as well as the aquaporins 1 and 5 (AQP1 and AQP5). Oxidative stress was assessed in bronchoalveolar lavage fluid (BALF).

**Results and discussion:**

This study demonstrates that UDCA and CDCA can mitigate endotoxin-induced lung injury in rats. These effects are achieved through modulation of AQP1 and AQP5 expression, reduction of oxidative stress, regulation of apoptotic pathways (BAX, caspase 3, BCL-2), and attenuation of pro-inflammatory activity of NF-κB. Although the results indicate a significant association between the expression of these proteins and histopathological changes, the potential influence of additional factors cannot be excluded. These findings suggest that UDCA and CDCA provide lung protection by acting through complex mechanisms involving inflammatory, oxidative, and apoptotic pathways.

## 1 Introduction

Acute lung injury (ALI) is one of the most common complications of sepsis. After the onset of systemic inflammatory response syndrome, systemically circulating mediators target the parenchyma or vasculature of the lungs, leading to tissue damage (Hasan et al., 2011). Pathophysiologically, ALI is characterized by damage of the alveolar-capillary barrier, interstitial and alveolar edema, neutrophil infiltration, and abnormal cell apoptosis (Wang et al., 2020; Li et al., 2016). The Toll-like receptor 4 (TLR4) plays a key role in the pathogenesis of ALI by recognizing the lipid components of endotoxin (lipopolysaccharide, LPS). TLR4 expression has been observed in various types of lung cells, including endothelial cells of blood vessels and epithelial cells of the respiratory tract (Baumgarten et al., 2006). It has been shown that the level of TLR4 expression is directly associated with the severity of the inflammatory response (Togbe et al., 2006).

ALI is characterized by the disturbance in the redox system’s balance, resulting in excessive reactive oxygen species (ROS) production, which plays a significant role in inflammation and the progression of lung damage in sepsis. Increased lipid peroxidation in plasma and reduced levels of antioxidants have been observed in patients with ALI (Abd Uljaleel and Hassan, 2023). Additionally, disruptions in the regulation of apoptotic pathways have been shown to contribute to epithelial and endothelial injuries, which are characteristic changes in ALI (Z΄graggen et al., 2010).

Some studies have highlighted the vital role of AQPs in the pathophysiology of pulmonary diseases. AQPs, also known as water channels, with a primary role in osmotically driven selective water transport into and out of the cell, across cell membranes of various organ systems (lungs, kidneys, etc.) (Yadav et al., 2020). Of the thirteen AQPs in the human body, AQPs 1, 3, 4, and 5 are expressed in the respiratory system (Bhattacharya et al., 2022). AQP1 and AQP5 are predominantly present in the endothelium of blood vessels and alveolar epithelium, facilitating fluid movement in the alveoli between the air space and associated vascular networks. AQPs are subject to modulation by various signaling pathways, with particular emphasis on AQP response in infection-related ALI (Zhang et al., 2018). The regulatory mechanisms of AQPs in endotoxemia appear to be tissue- and AQP-specific. AQP expression seems to change in different pathological mechanisms of sepsis and may be crucial in inflammation. Additionally, the role of a specific AQP in an endotoxin-induced ALI model depends on the site of injury, i.e., whether it involves the epithelium or endothelium (Rump and Adamzik, 2018).

Given that the lungs are one of the most frequently affected organs during sepsis, it is necessary to initiate a series of studies to gain a deeper understanding of the fundamental pathophysiological mechanisms and develop new therapeutic strategies for ALI. A potentially promising therapeutic approach could be based on the role of bile acids. Contemporary understanding of bile acids emphasizes their hormonal status and role in regulating metabolic processes through the activation of signaling pathways via membrane and nuclear bile acid receptors, present in many organs and cells, including lung cells, with varying levels of expression (Pols et al., 2011). This complex function makes them the key metabolic and immunological regulators, expanding the understanding of their function beyond the traditional role in digestion and absorption (Staels and Fonseca, 2009). Reports on the wide range of protective effects of ursodeoxycholic acid (UDCA), recognized as the safest among bile acids, have also been demonstrated in ALI (Niu et al., 2020). The structural isomer of UDCA, chenodeoxycholic acid (CDCA), has demonstrated cytotoxic effects in several studies (Gong et al., 2016), but there are also studies indicating the cytoprotective effects of CDCA (Han, 2020), including its protective role in lung damage (Aldhahrani et al., 2017).

We hypothesize that pretreatment with bile acids may affect the lung injury, so the aim of this study was to elucidate the impact of UDCA and CDCA on oxidative stress, inflammation, and apoptosis in endotoxin-induced ALI in rats.

## 2 Material and methods

### 2.1 Experimental animals

Thirty-six male *Wistar albino* rats were housed in groups of six per cage under controlled laboratory conditions, including a room temperature of 21°C ± 2°C, a 12-h light/dark cycle, and 55% ± 5% humidity, with unlimited access to food and water. The rats were given a 1-week acclimatization period before beginning the experiments. All experimental animals, protocols, and procedures were approved by the Ethics Committee for the Protection of Welfare of Experimental Animals at the Faculty of Medicine, University of Banja Luka (approval number 18/1.190–1/22, dated 9 March 2022).

### 2.2 Experimental groups

**FIGURE 1:**
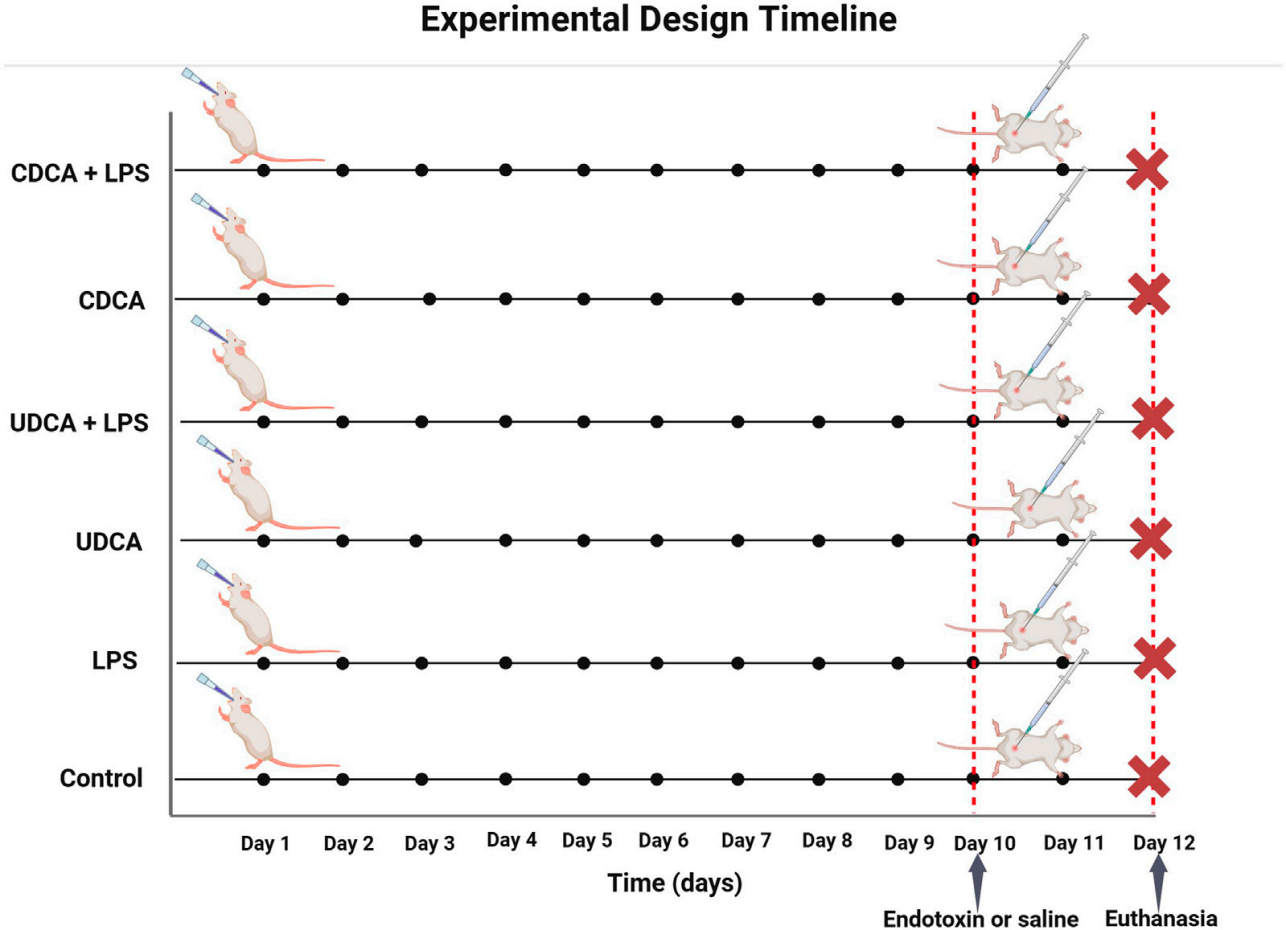
Schematic representation of the experimental design with time points and groups.

The animals were assigned to six distinct groups, each comprising six individuals. They underwent a pretreatment regimen involving either propylene glycol, UDCA, or CDCA for 10 days. The Control (C) group received 0.5 mL/kg of propylene glycol orally (p.o.) for 10 days, and on the 10th day, they were administered 1 mL/kg of saline intraperitoneally (i.p.). The LPS group was treated with 0.5 mL/kg of propylene glycol p.o. for 10 days and 5.5 mg/kg of endotoxin i.p. on the 10th day. The UDCA group was given 25 mg/kg of UDCA p.o. for 10 days, followed by 1 mL/kg of saline i.p. on the 10th day. The UDCA + LPS group received UDCA in the same dosage and manner as the UDCA group, but on the 10th day, they were administered 5.5 mg/kg of endotoxin i.p. The CDCA group was administered 25 mg/kg of CDCA p.o. for 10 days, and on the 10th day, they received 1 mL/kg of saline i.p. The CDCA + LPS group received CDCA in the same dosage and manner as the CDCA group, with the addition of 5.5 mg/kg of endotoxin i.p. on the 10th day (Figure 1).

### 2.3 Bronchoalveolar lavage sampling

After the animals were anesthetized, their tracheas were exposed, and a cannula was inserted via a catheter. Bronchoalveolar lavage fluid (BALF) was collected by instilling and aspirating 0.7 mL of cold phosphate-buffered saline (PBS) three times. The collected samples were centrifuged at 5,000 rpm at 4°C for 10 min, and the supernatant was separated for the analysis of oxidative stress parameters. All samples were stored at −80°C until further measurement. The parameters of oxidative stress assessed in BALF included superoxide dismutase (SOD), catalase (CAT), glutathione (GSH), thiobarbituric acid reactive substances (TBARS), and nitrites (NO_2_^−^). Pro-oxidative markers TBARS, indicative of lipid peroxidation, and nitrites, were quantified spectrophotometrically using a Shimadzu UV 1800 device from Japan (Ohkawa et al., 1979; Green et al., 1982). The activity of SOD and CAT in BALF was determined using the methods of Madesh and Balasubramanian (1998) and Aebi (1984), respectively, and the level of GSH was determined using Ellman’s method (Eftekhar et al., 2018).

### 2.4 Tissue collection

At the end of the experiment, the rats were anaesthetized with a mixture of ketamine (90 mg/kg) and xylazine (10 mg/kg). Their lungs were then excised and after fixation in 10% neutral buffered formalin, the tissue samples were processed in an automatic tissue processor (Leica TP1020, Leica Biosystems United States). Following a standard protocol, the tissues were dehydrated in increasing concentrations of ethyl alcohol (70%, 96%, 100%), then cleared in xylene (three changes), impregnated with liquid paraffin, and embedded in paraffin blocks. For histological and immunohistochemical analysis, the paraffin blocks were sectioned at a thickness of 4–5 µm using a rotary microtome (Rotary 3003 pfm, pfmmedical, Germany). The resulting sections were placed on appropriate slides and dried at 60°C.

### 2.5 Histopathological analysis

In the first step of the histological procedure, tissue sections were deparaffinized in xylene (two changes of 5 min each), then rehydrated in decreasing concentrations of ethyl alcohol (100%, 96%, and 70%, 5 min each) and rinsed with distilled water. Following this, the sections were stained using the standard haematoxylin-eosin (H&E) method and analysed with a Leica DM2500 light microscope (Leica Microsystems, Switzerland).

To assess histological changes indicating lung damage, observed by two independent pathologists in a blinded manner, scoring from 1 to 4 was performed: Score 1 - no pathological changes in the lungs; Score 2 - mild lung tissue damage, with multifocal degeneration and mild inflammatory infiltration or focal damage to lung cells; Score 3 - moderate lung tissue damage, with severe tissue degeneration and/or diffuse inflammation; Score 4 - severe lung tissue damage, necrosis with diffuse inflammation. Lung sections were scored, and the average histopathological score per group was calculated.

### 2.6 Immunohistochemical analysis

For immunohistochemical analysis, a standard protocol was employed. Tissue sections from the lungs were deparaffinized and rehydrated. Slides underwent a 20-min boiling process in a PT module (Pretreatment Module™, Lab Vision Corporation, United States) with citric acid buffer solution (0.01 mol/L citrate buffer, pH 6.0). To minimize nonspecific background staining, slides were treated with 3% hydrogen peroxide for 10 min. Primary antibodies at the appropriate concentration were applied to the tissues and incubated overnight at 4°C in a humid chamber. Samples were incubated with following primary antibodies: NF-κB (ab16502, dilution 1:100), BCL-2 (ab196495, dilution 1:200), cleaved caspase-3 (Asp175, dilution 1:200), BAX (ab32503, dilution 1:300), AQP1 (sc-25287, dilution 1:75), and AQP5 (sc-514022, dilution 1:50). The specificity of the interaction between the primary antibodies and their antigenic determinants was revealed using sensitive detection systems composed of secondary anti-mouse and/or anti-rabbit IgG conjugated with horseradish peroxidase (HRP). A ready-to-use polyvalent, polymeric detection system was used (UltraVision Detection System HRP Polymer & DAB Plus Chromogen, Thermo Fisher Scientific, United States). The antibody binding sites in the tissue became visible after brief staining (5 min) with 3,3′-diaminobenzidine tetrachloride (DAB) chromogen, which produces a brown precipitate at the site of a positive reaction. Subsequently, all sections were stained using the hematoxylin, dehydrated, and mounted. Appropriate positive and negative controls were concurrently processed.

After immunohistochemical staining, the microscopic slides were examined under a Leica DM2500 light microscope equipped with an MC170HD digital camera (Leica Microsystems, Switzerland). Microphotographs were captured at ×400 magnification under identical optical conditions and archived in ‘Tiff’ format (2592 × 1944 pixels). Photometric analysis of the immunohistochemical staining results was performed using the Fiji software for biological image analysis (National Institutes of Health, Bethesda, MD, United States [version 1.54f]), utilizing the color deconvolution option to extract the matrix from the microphotographs with specifically bound immunohistochemical chromogen–DAB. The software-based photometry determined the tissue fraction areas stained with DAB, the mean optical density of DAB, as well as the percentage of DAB (% DAB) in the rat lung tissue fraction. Data are presented as the mean optical density ±standard deviation and % DAB ±standard deviation in the rat lung tissue fraction. The average mean optical density and % DAB were compared between groups.

### 2.7 Statistical analysis

Statistical analysis was performed with IBM-SPSS Statistic version 20.0 software (SPSS, Inc., Chicago IL, United States). ANOVA test was used to compare the means of parametric characteristics, while Kruskal–Wallis and Mann-Whitney U tests are used to compare the nonparametric characteristics between the groups. Tukey and Bonferroni tests are used for *post hoc* analysis. Linear correlation was used to explore the relationship between optical density and % DAB in lung tissue fractions. Results are presented as mean ± standard deviation or median ± interquartile range. Differences between groups were considered significant at a p-value < 0.05.

## 3 Results

### 3.1 Effects of UDCA and CDCA pretreatment on oxidative stress markers measured in BALF in endotoxin-induced ALI

**FIGURE 2:**
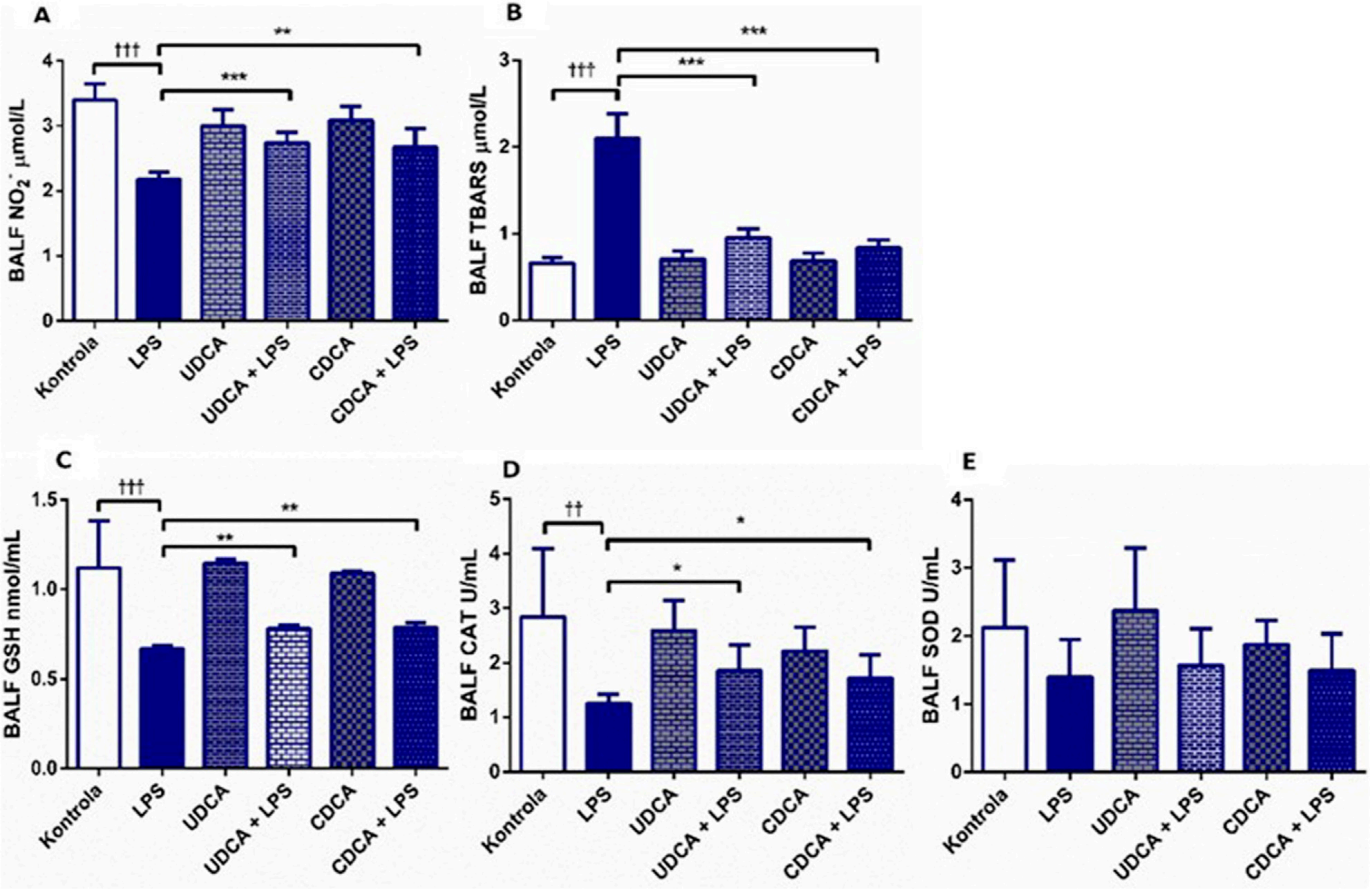
Effects of UDCA and CDCA administration on oxidative stress markers in BALF. **(A)** TBARS level; **(B)** NO2- level; **(C)** GSH level; **(D)** CAT activity; **(E)** SOD activity. Data are expressed as mean ± SD or mean ± SE. LPS-group treated with endotoxin; UDCA-group treated with ursodeoxycholic acid; UDCA + LPS-group treated with ursodeoxycholic acid and endotoxin; CDCA-group treated with chenodeoxycholic acid; CDCA + LPS-group treated with chenodeoxycholic acid and endotoxin. One-way ANOVA, Bonferroni test, and Mann-Whitney test were performed. Asterisk (*) denotes significant differences compared to the LPS group *p < 0.05, ***p < 0.001, and dagger (†) denotes significant differences compared to the C group †*p* < 0.05; ††*p* < 0.01; †††*p* < 0.001.

In ALI induced by endotoxin administration, UDCA and CDCA showed antioxidant effects (Figure 2). Endotoxin caused a decrease in NО_2_^-^ levels (p < 0.001) compared to the control, and the application of UDCA and CDCA significantly attenuated this effect (p < 0.001, p < 0.01, respectively) (Figure 2A). TBARS, a marker of lipid peroxidation, showed a significant increase in the endotoxin-treated group compared to the control group (p < 0.001), indicating the involvement of endotoxin in oxidative stress in ALI. The application of UDCA and CDCA significantly reduced TBARS levels (p < 0.001) (Figure 2B). GSH, CAT, and SOD were examined as components of the antioxidant defense. GSH and CAT showed a decrease in the endotoxin-treated group (p < 0.001, p < 0.01, respectively), and pretreatment with UDCA and CDCA demonstrated their antioxidant effects by increasing GSH levels (p < 0.01) and CAT activity (p < 0.05) (Figures 2C, D), but not for SOD (Figure 2E).

### 3.2 Effects of UDCA and CDCA pretreatment on lung histopathological characteristics in endotoxin-induced ALI

**FIGURE 3:**
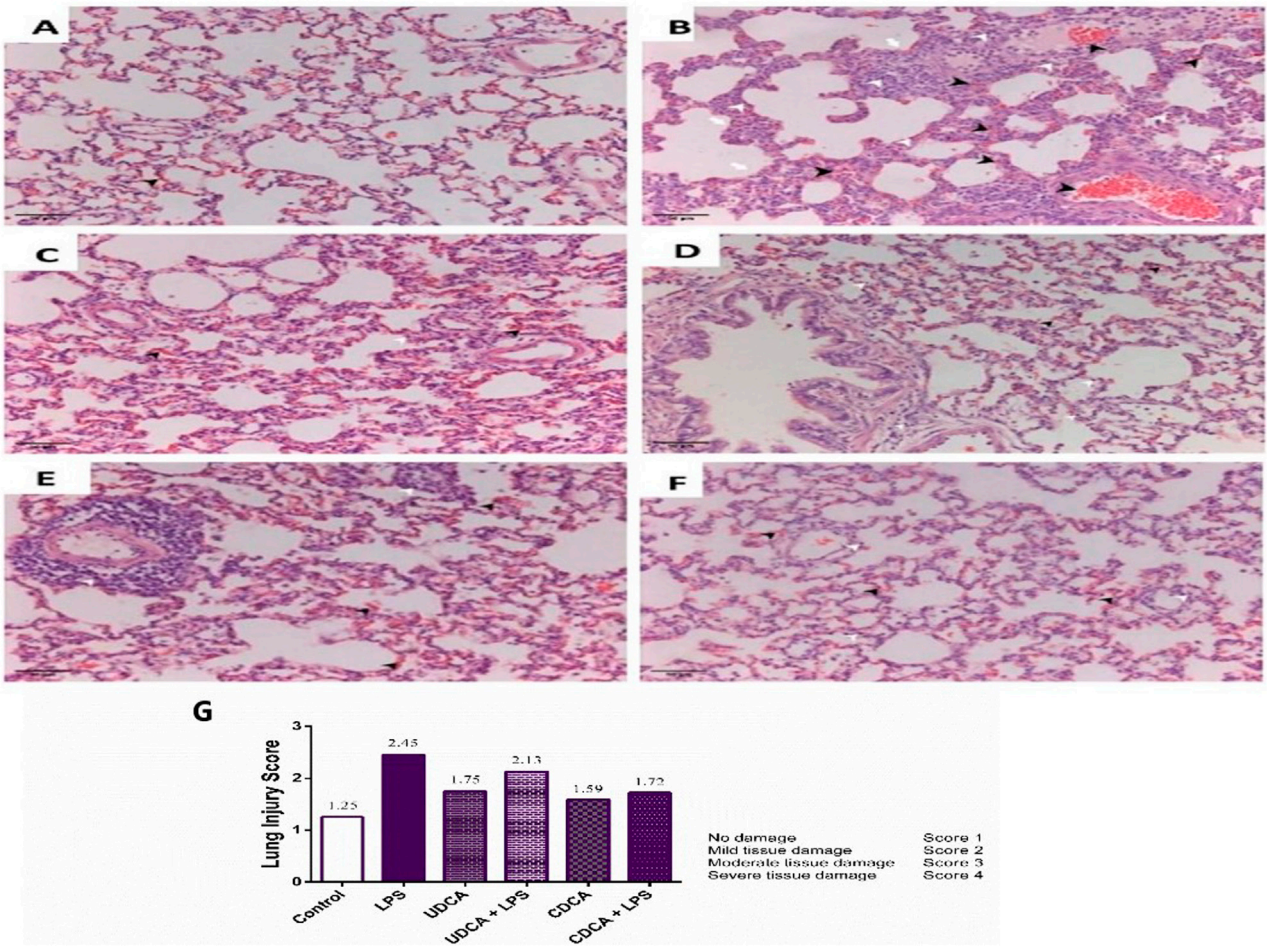
Lung specimen (H&E staining). Magnification ×20 and Lung injury score. **(A)** The control group. The sample shows preserved lung tissue with mild extravasation. **(B)** Endotoxin-treated group. The sample exhibits acute lung parenchymal injury characterized by hemorrhage/extravasation, blood vessels congestion, interstitial bleeding (indicated by black arrowhead), inflammatory infiltrate (indicated by white arrowhead), and alveolar wall thickening (indicated by white arrow). Vascular congestion and perivascular inflammatory infiltrate are evident. **(C)** UDCA group. The sample shows preserved lung parenchyma with moderate extravasation and interstitial inflammatory infiltrate. **(D)** UDCA + LPS group. The sample demonstrates preserved lung parenchyma with reduced interstitial extravasation and inflammatory infiltrate. Vascular walls are preserved without luminal congestion. **(E)** CDCA group. The sample displays preserved lung parenchyma with extravasation. There is predominantly perivascular localized inflammatory infiltrate. **(F)** CDCA + LPS group. The sample shows preserved lung parenchyma with reduced extravasation and predominantly perivascular interstitial inflammatory infiltrate. Vascular walls remain preserved without luminal congestion. **(G)** Lung injury score. Data are expressed as the median ± IQR. The lung damage score was calculated based on haematoxylin-eosin (H&E) stained images. LPS group–treated with endotoxin; UDCA group–treated with ursodeoxycholic acid; UDCA + LPS group–treated with ursodeoxycholic acid and endotoxin; CDCA group–treated with chenodeoxycholic acid; CDCA + LPS group–treated with chenodeoxycholic acid and endotoxin.

Our study showed normal lung morphology in the control group (Figure 3A). The application of UDCA and CDCA did not damage the lung structure; the pulmonary parenchyma was preserved (Figures 3C, E). The endotoxin-treated group showed significant lung tissue damage compared to the control group, with visible interstitial hemorrhage, inflammation, and thickening of the interalveolar septa, vascular congestion, and perivascular inflammatory infiltrate (Figure 3B). The application of UDCA and CDCA alleviated endotoxin-induced lung damage. In these rats, a reduction in interstitial extravasation and inflammatory infiltrate was observed, with preserved vascular walls without luminal congestion (Figures 3D, F), indicating the effectiveness of pretreatment with UDCA and CDCA in reducing endotoxin-induced lung injury.

A semi-quantitative pathological score based on the severity of tissue damage is presented in Figure 3G A score of one indicates physiological tissue characteristics. Higher scores indicate different levels of tissue damage (Figure 3G). The reduction in the lung damage score in the groups of rats treated with UDCA and CDCA indicated their potential protective effect in endotoxin-induced ALI.

### 3.3 Correlations between optical density and % DAB in lung tissue fractions

The results of the optical density values and % DAB in lung tissue fractions, including the biomarkers NF-κB, BCL-2, caspase-3, BAX, AQP1, and AQP5 are presented in Table 1. There are statistically significant correlations between optical density and % DAB for BCL-2, caspase-3, BAX, and AQP1 (p < 0.001), while for NF-κB and AQP5 a correlation was observed at a significance level of p < 0.01.

**TABLE 1:** Data values and correlations between optical density and % DAB in lung tissue fractions.

		Control Mean ± SD	LPS Mean ± SD	UDCA Mean ± SD	UDCA + LPS Mean ± SD	CDCA Mean ± SD	CDCA + LPS Mean ± SD	r
NfkB	% DAB	41.35 ± 13.70	70.4 ± 14.22	34.05 ± 16.43	67.55 ± 17.66	44.25 ± 17.30	42.35 ± 5.82	0.331**
	Optical density	0.61 ± 0.07	1.04 ± 0.33	0.58 ± 0.09	0.59 ± 0.12	0.63 ± 0.05	0.74 ± 0.12	
BCL2	% DAB	20.4 ± 9.92	9.9 ± 5.29	20.65 ± 5.98	28.15 ± 8.98	26.05 ± 7.34	28.35 ± 5.51	0.497***
	Optical density	0.70 ± 0.13	0.47 ± 0.08	0.65 ± 0.08	0.73 ± 0.13	0.82 ± 0.07	0.82 ± 0.09	
CC3	% DAB	34.00 ± 12.92	68.75 ± 13.83	33.45 ± 10.45	39.10 ± 16.04	33.90 ± 9.77	28.40 ± 5.92	0.652***
	Optical density	0.64 ± 0.13	1.43 ± 0.39	0.68 ± 0.17	0.55 ± 0.10	0.58 ± 0.15	0.70 ± 0.11	
BAX	% DAB	19.65 ± 7.55	60.0 ± 13.5	24.75 ± 9.31	27.15 ± 8.02	24.8 ± 7.84	21.9 ± 10.14	0.680***
	Optical density	0.72 ± 0.14	1.08 ± 0.32	0.85 ± 0.11	0.76 ± 0.06	0.72 ± 0.06	0.74 ± 0.09	
AQP1	% DAB	54.12 ± 11.79	14.75 ± 6.09	33.02 ± 6.74	33.05 ± 9.64	46.54 ± 12.35	33.02 ± 13.14	0.458***
	Optical density	0.95 ± 0.16	0.63 ± 0.11	0.85 ± 0.12	0.98 ± 0.16	0.92 ± 0.10	0.92 ± 0.12	
AQP5	% DAB	36.88 ± 14.62	11.35 ± 7.42	25.58 ± 11.50	37.33 ± 14.64	44.31 ± 16.48	34.43 ± 14–39	0.282**
	Optical density	0.82 ± 0.18	0.51 ± 0.14	0.70 ± 0.15	0.71 ± 0.23	0.88 ± 0.24	0.76 ± 0.23	

LPS, group–treated with endotoxin; UDCA, group–treated with ursodeoxycholic acid; UDCA + LPS, group–treated with ursodeoxycholic acid and endotoxin; CDCA, group–treated with chenodeoxycholic acid; CDCA + LPS, group–treated with chenodeoxycholic acid and endotoxin. % DAB-3, 3′-diaminobenzidine tetrachloride in tissue fraction; SD- Standard deviation. r-Pearson’s correlation factor. An asterisk (*) indicates significant of correlation between OD, and % DAB, in lung tissue fraction, ***p < .001; **p < .01.

### 3.4 Effects of UDCA and CDCA pretreatment on NF-κB expression in endotoxin-induced ALI

**FIGURE 4:**
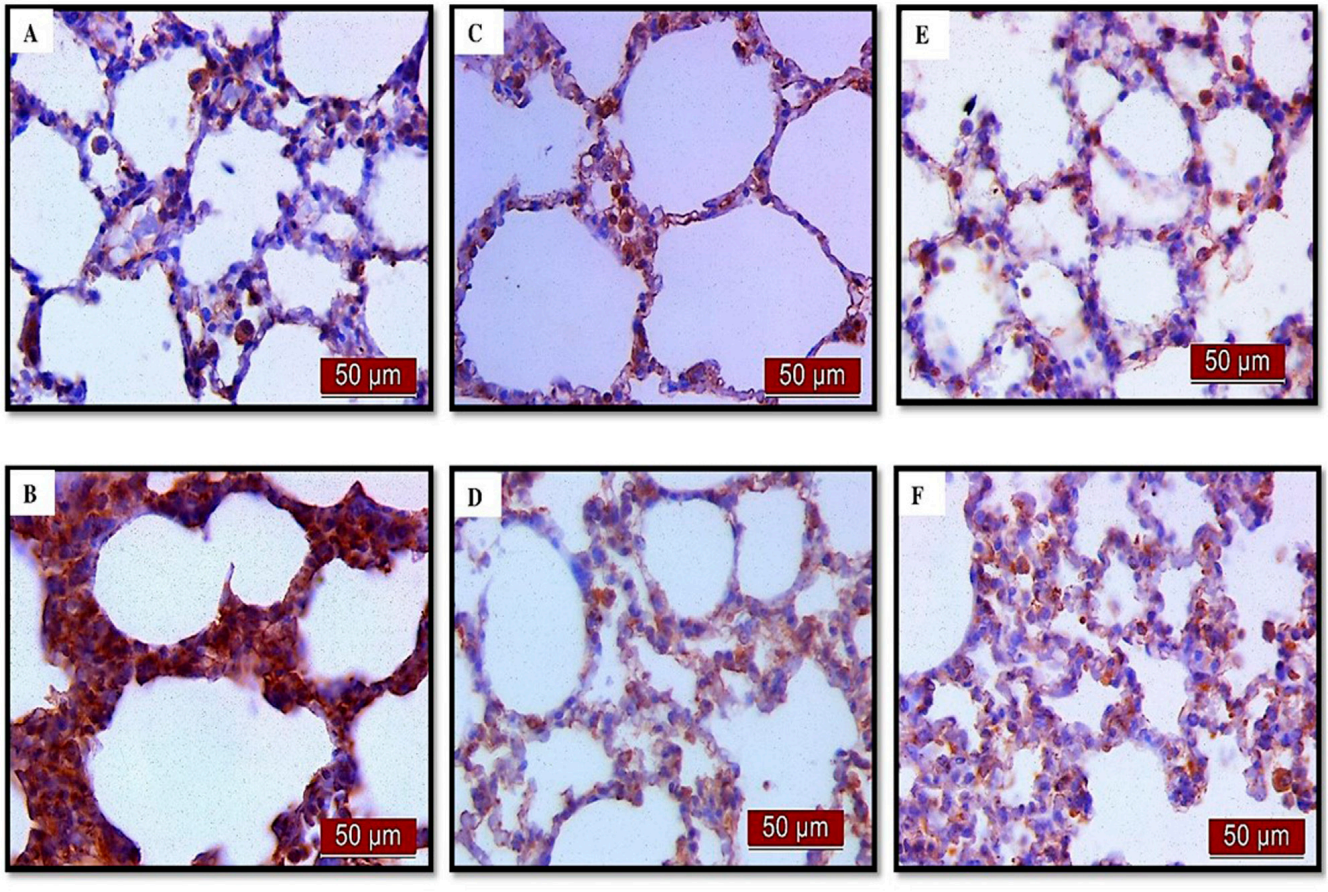
The distribution of lung NF-κB expression detected by immunohistochemistry (×400 magnification). **(A)** The control group did not show NF-κB expression. **(B)** The LPS group showed a significant increase in NF-κB immunoreactivity in alveolar parenchyma. The brown color indicates NF-κB positivity. **(C)** The UDCA group did not show NF-κB expression. **(D)** The UDCA + LPS group showed a significant reduction in NF-κB immunostaining. **(E)** The CDCA group also did not show NF-κB expression. **(F)** The CDCA + LPS group showed a significant reduction in NF-κB immunostaining.

**FIGURE 5:**
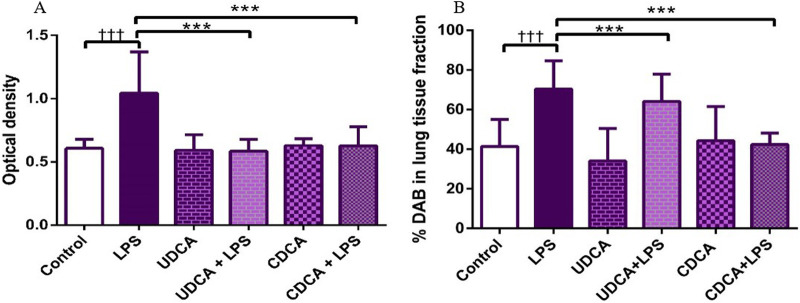
The value of NF-κB immunohistochemically positive areas in rat lung tissue in different groups. **(A)** The optical density **(B)** % DAB in rat lung tissue fraction. LPS group–treated with endotoxin; UDCA group–treated with ursodeoxycholic acid; UDCA + LPS group–treated with ursodeoxycholic acid and endotoxin; CDCA group–treated with chenodeoxycholic acid; CDCA + LPS group–treated with chenodeoxycholic acid and endotoxin. One-way ANOVA, Bonferroni test, and Mann-Whitney test were performed. An asterisk (*) indicates significant differences compared to the LPS group ***p < 0.001, while the symbol (†) indicates significant differences compared to the control group †††*p* < 0.001.

(A) Immunohistochemical assessment of NF-κB (Figure 4) (B) The optical density (Figure 5A), and % DAB (Figure 5B) in rat lung tissue fraction of NF-κB showed a significant increase in NF-κB expression in alveolar parenchyma in rat lungs after endotoxin administration compared to the control group (p < 0.001). Rats pretreated with UDCA and CDCA demonstrated a significant reduction in endotoxin-induced overexpression of NF-κB (p < 0.001). Rats that received UDCA or CDCA, similar to the control group, had negative immunohistochemical findings for NF-κB. These results strongly indicate a significant reduction in NF-κB expression in endotoxin-induced lung injury through the administration of UDCA and CDCA.

### 3.5 Effects of UDCA and CDCA pretreatment on apoptosis markers in endotoxin-induced ALI

**FIGURE 6:**
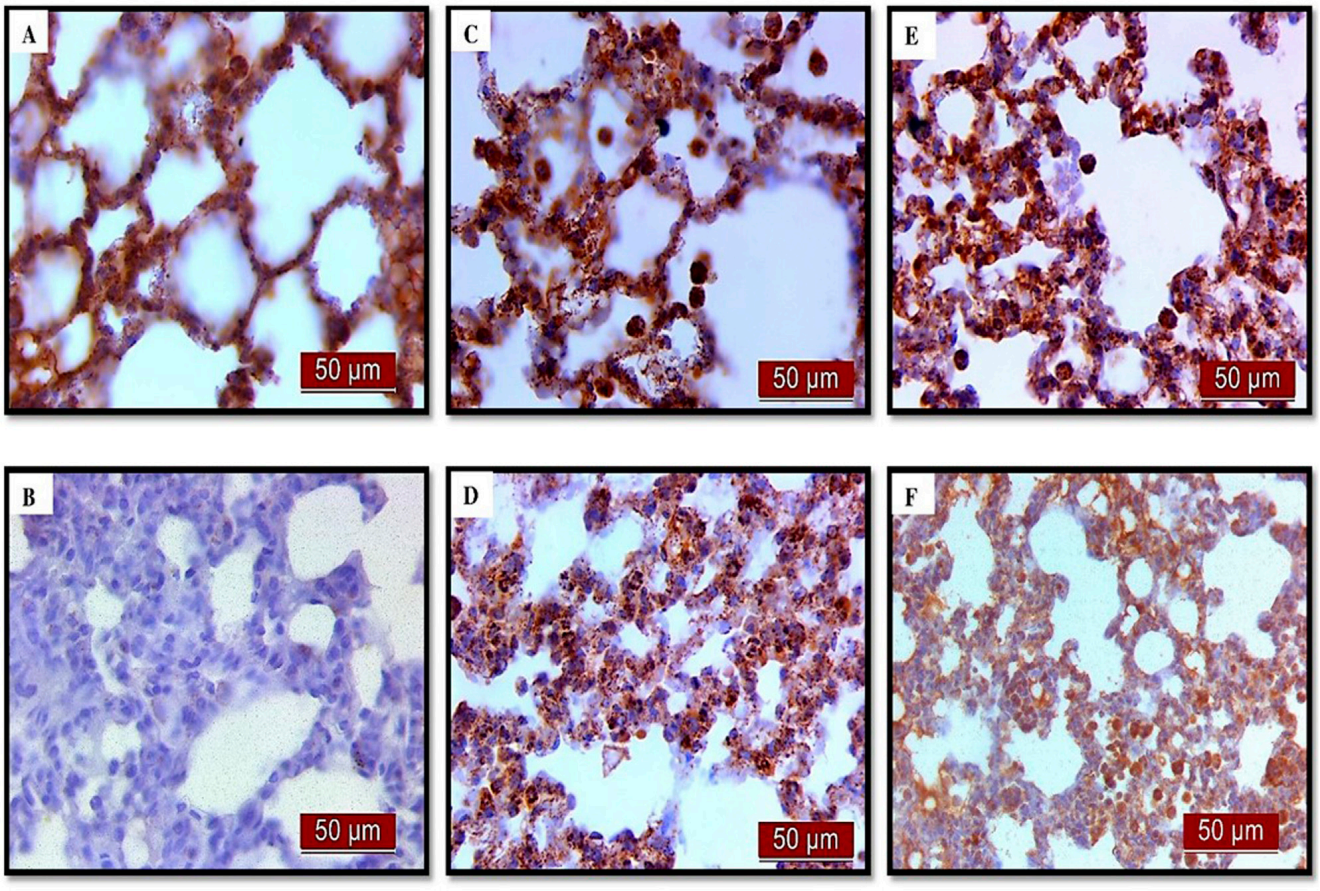
The distribution of lung BCL-2 expression detected by immunohistochemistry (×400 magnification). **(A)** The control group showed significant BCL-2 expression. **(B)** The LPS group showed a significant decrease in BCL-2 immunoreactivity in alveolar parenchyma. Brown color indicates BCL-2 positivity. **(C)** The UDCA group showed BCL-2 expression. **(D)** The UDCA + LPS group showed a significant increase in BCL-2 immunostaining. **(E)** The CDCA group also showed BCL-2 expression. **(F)** The CDCA + LPS group showed a significant increase in BCL-2 immunostaining.

**FIGURE 7:**
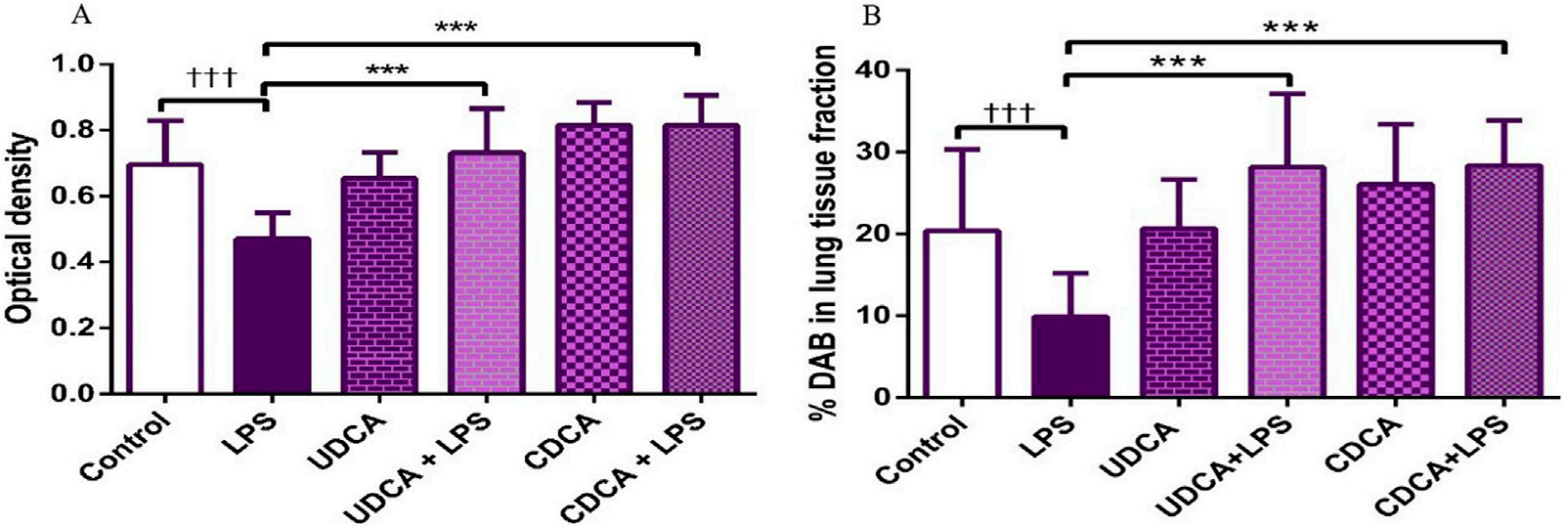
The value of BCL2 immunohistochemically positive areas in rat lung tissue in different groups. **(A)** The optical density **(B)** % DAB in rat lung tissue fraction. LPS group–treated with endotoxin; UDCA group–treated with ursodeoxycholic acid; UDCA + LPS group–treated with ursodeoxycholic acid and endotoxin; CDCA group–treated with chenodeoxycholic acid; CDCA + LPS group–treated with chenodeoxycholic acid and endotoxin. One-way ANOVA, Bonferroni test, and Mann-Whitney test were performed. An asterisk (*) indicates significant differences compared to the LPS group ***p < 0.001, while the symbol (†) indicates significant differences compared to the control group †††*p* < 0.001.

Immunohistochemical analysis revealed a significant decrease in BCL-2 immunoreactivity in alveolar parenchyma in rat lungs treated with endotoxin, while an increase in immunoreactivity was observed in the UDCA and CDCA-treated groups (Figure 6). The lowest optical density value (Figure 7A), and % DAB (Figure 7B) of BCL-2 found in the endotoxin-treated group (p < 0.001), whereas UDCA and CDCA significantly increased the optical density and % DAB of BCL-2, demonstrating protective effects (p < 0.001) (Figures 7A, B).

**FIGURE 8:**
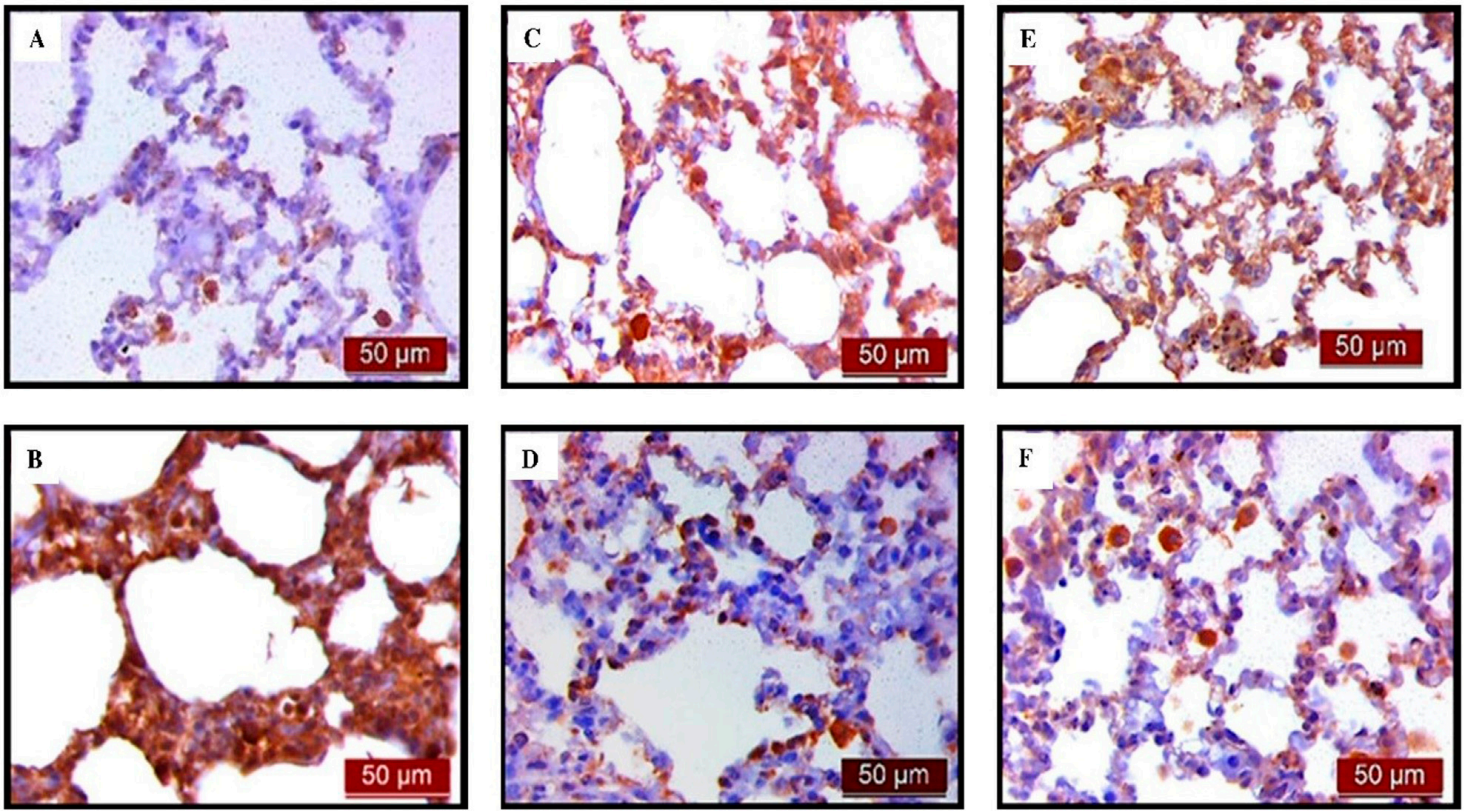
The distribution of lung caspase three expression detected by immunohistochemistry (×400 magnification). **(A)** The control group did not show caspase three expression. **(B)** The LPS group showed a significant increase in caspase three immunoreactivity in alveolar parenchyma. The brown colour indicates caspase three positivity. **(C)** The UDCA group did not show caspase three expression. **(D)** The UDCA + LPS group showed a significant reduction in caspase three immunostaining. **(E)** The CDCA group also did not show caspase three expression. **(F)** The CDCA + LPS group showed a significant reduction in caspase three immunostaining.

**FIGURE 9:**
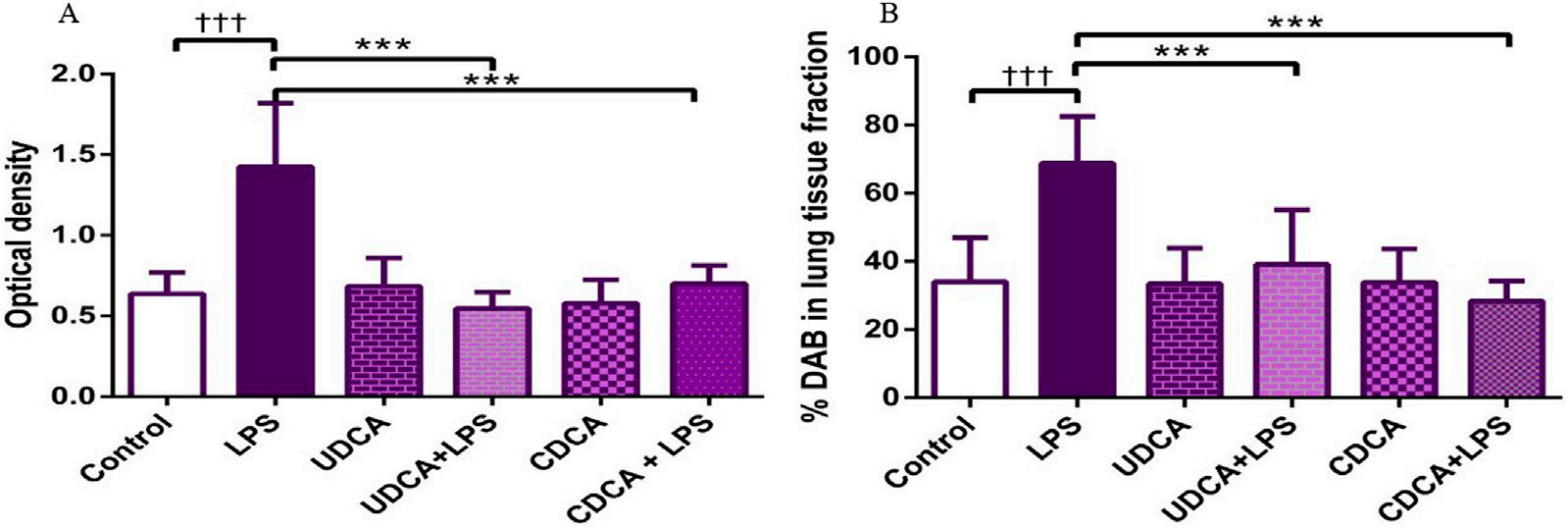
The value of caspase three immunohistochemically positive areas in rat lung tissue in different groups. **(A)** The optical density **(B)** % DAB in rat lung tissue fraction. LPS group–treated with endotoxin; UDCA group–treated with ursodeoxycholic acid; UDCA + LPS group–treated with ursodeoxycholic acid and endotoxin; CDCA group–treated with chenodeoxycholic acid; CDCA + LPS group–treated with chenodeoxycholic acid and endotoxin. One-way ANOVA, Bonferroni test, and Mann-Whitney test were performed. An asterisk (*) indicates significant differences compared to the LPS group ***p < 0.001, while the symbol (†) indicates significant differences compared to the control group †††*p* < 0.001.

The results of the analysis of the pro-apoptotic marker caspase three showed that UDCA and CDCA pretreatment significantly decreased (p < 0.001) caspase three expression in endotoxin-induced ALI. Figure 8 displays the immunohistochemical staining of caspase three in alveolar parenchyma in rat lungs across all experimental groups, while Figures 9A, B shows the optical density value and % DAB of caspase 3.

**FIGURE 10:**
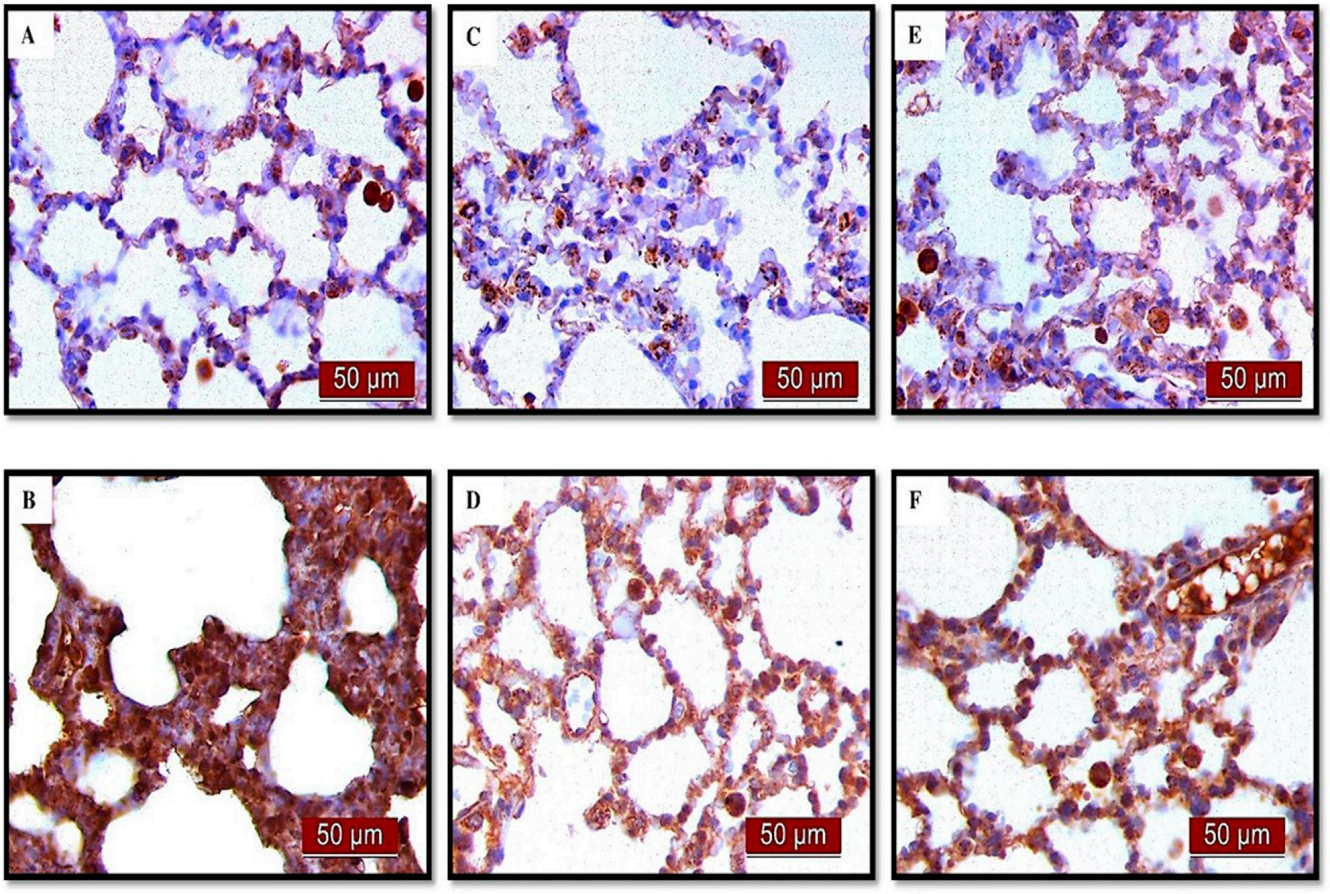
The distribution of lung BAX expression detected by immunohistochemistry (×400 magnification) **(A)** The control group did not show BAX expression. **(B)** The LPS group showed a significant increase in BAX immunoreactivity in alveolar parenchyma. The brown color indicates BAX positivity. **(C)** The UDCA group did not show BAX expression. **(D)** The UDCA + LPS group showed a significant reduction in BAX immunostaining. **(E)** The CDCA group also did not show BAX expression. **(F)** The CDCA + LPS group showed a significant reduction in BAX immunostaining.

**FIGURE 11:**
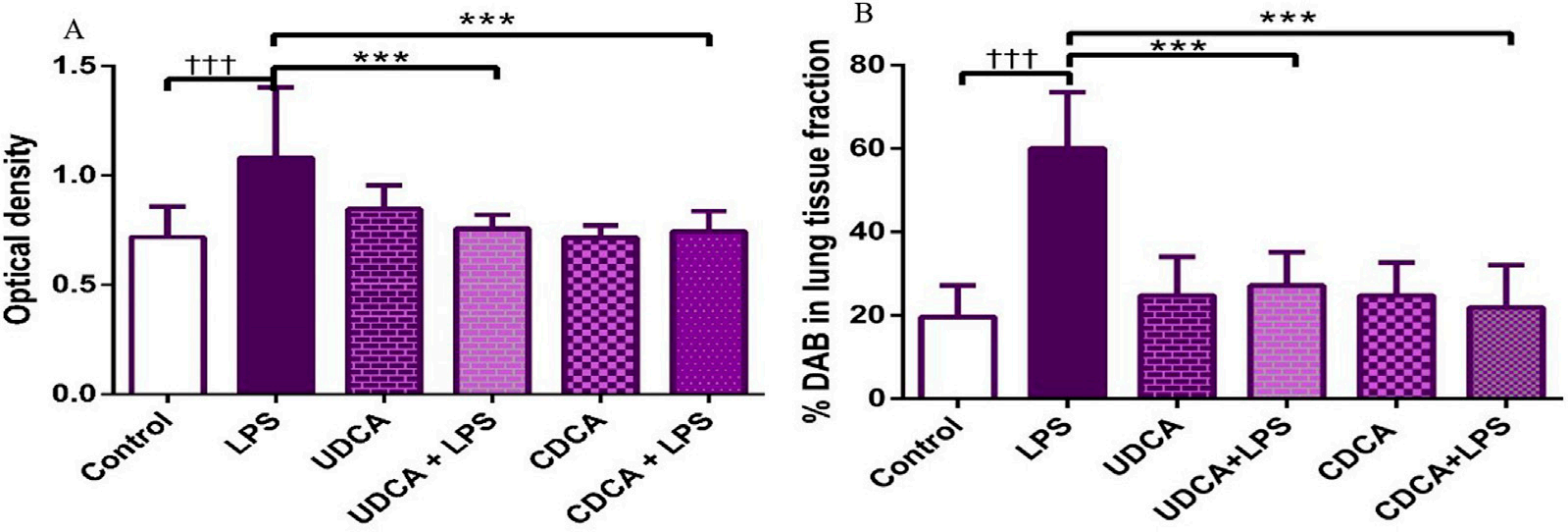
The value of BAX immunohistochemically positive areas in rat lung tissue in different groups. **(A)** The optical density **(B)** % DAB in rat lung tissue fraction. LPS group–treated with endotoxin; UDCA group–treated with ursodeoxycholic acid; UDCA + LPS group–treated with ursodeoxycholic acid and endotoxin; CDCA group–treated with chenodeoxycholic acid; CDCA + LPS group–treated with chenodeoxycholic acid and endotoxin. One-way ANOVA, Bonferroni test, and Mann-Whitney test were performed. An asterisk (*) indicates significant differences compared to the LPS group ***p < 0.001, while the symbol (†) indicates significant differences compared to the control group †††*p* < 0.001.

Figures 10, 11A present the results of the analysis of the pro-apoptotic marker BAX in alveolar parenchyma in rat lungs, which showed a significant increase in immunoreactivity in the endotoxin-treated group (p < 0.001). In contrast, UDCA and CDCA exhibited a protective effect by significantly reducing the expression of this pro-apoptotic marker (p < 0.001).

### 3.6 Effects of UDCA and CDCA pretreatment on the expression of AQP1 and AQP5 in endotoxin-induced ALI

**FIGURE 12:**
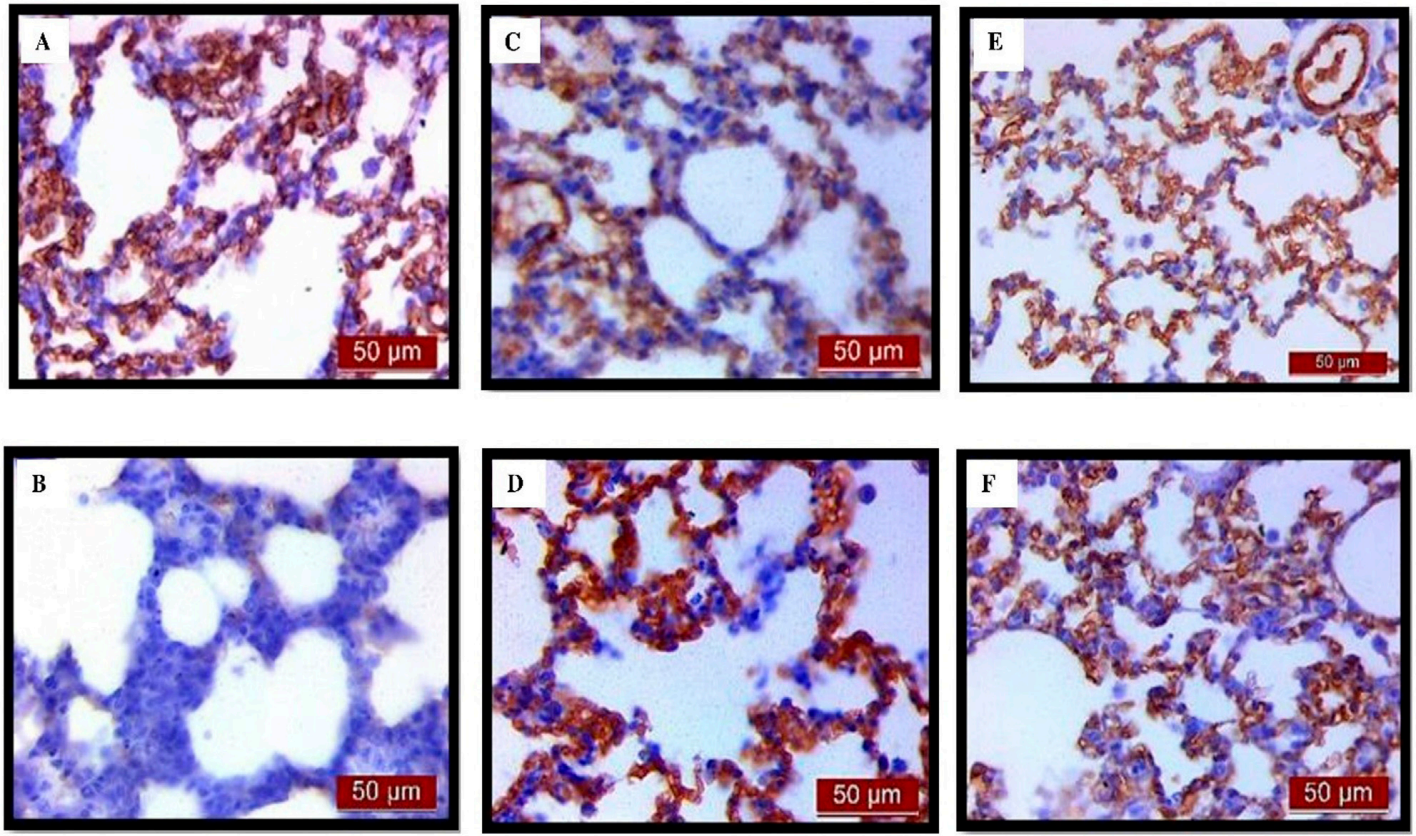
The distribution of lung AQP1 expression detected by immunohistochemistry (×400 magnification). **(A)** The control group showed significant AQP1 expression. **(B)** The LPS group showed a significant decrease in AQP1 immunoreactivity in alveoli in rat lungs. The brown color indicates AQP1 positivity. **(C)** The UDCA group showed AQP1 expression. **(D)** The UDCA + LPS group showed a significant increase in AQP1 immunostaining. **(E)** The CDCA group also showed AQP1 expression. **(F)** The CDCA + LPS group showed a significant increase in AQP1 immunostaining.

**FIGURE 13:**
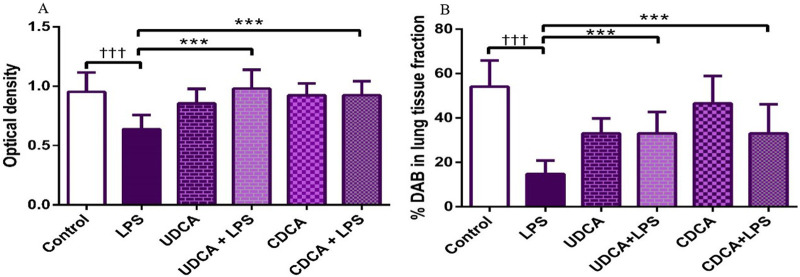
The value of AQP1 immunohistochemically positive areas in rat lung tissue in different groups. **(A)** The optical density **(B)** % DAB in rat lung tissue fraction. LPS group–treated with endotoxin; UDCA group–treated with ursodeoxycholic acid; UDCA + LPS group–treated with ursodeoxycholic acid and endotoxin; CDCA group–treated with chenodeoxycholic acid; CDCA + LPS group–treated with chenodeoxycholic acid and endotoxin. One-way ANOVA, Bonferroni test, and Mann-Whitney test were performed. An asterisk (*) indicates significant differences compared to the LPS group ***p < 0.001, while the symbol (†) indicates significant differences compared to the control group †††*p* < 0.001.

Immunohistochemical analysis revealed a significant decrease in AQP1 and AQP5 immunoreactivity in alveolar parenchyma in rat lungs in the endotoxin-treated group and an increase in UDCA and CDCA pretreated groups (Figure 12). Additionally, the lowest optical density value and % DAB of AQP1 was present in the endotoxin-treated group (p < 0.001). Both bile acids, UDCA and CDCA, again demonstrated protective effects in endotoxin-induced ALI by significantly increasing the optical density value and % DAB of AQP1 in rat lung tissue (p < 0.001) (Figures 13A, B).

**FIGURE 14:**
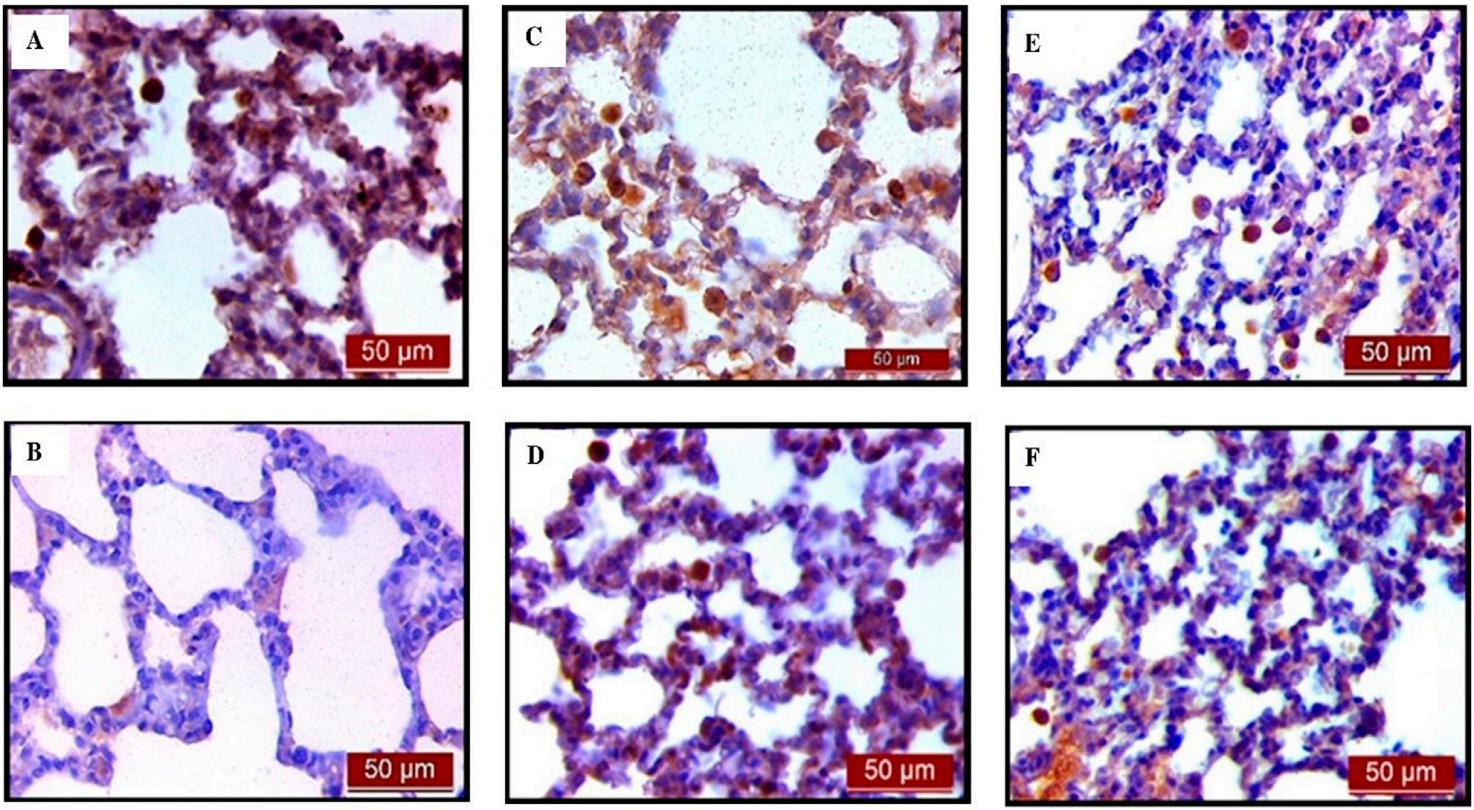
The distribution of lung AQP5 expression detected by immunohistochemistry (×400 magnification). **(A)** The control group showed significant AQP5 expression. **(B)** The LPS group showed a significant decrease in AQP5 immunoreactivity in alveolar parenchyma. The brown color indicates AQP5 positivity. **(C)** The UDCA group showed AQP5 expression. **(D)** The UDCA + LPS group showed a significant increase in AQP5 immunostaining. **(E)** The CDCA group also showed AQP5 expression. **(F)** The CDCA + LPS group showed a significant increase in AQP5 immunostaining.

**FIGURE 15:**
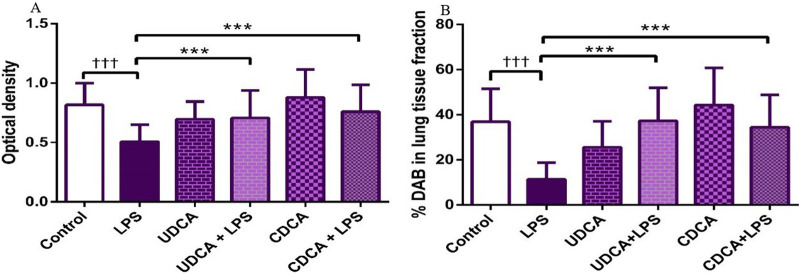
The value of AQP5 immunohistochemically positive areas in rat lung tissue in different groups. **(A)** The optical density **(B)** % DAB in rat lung tissue fraction. LPS group–treated with endotoxin; UDCA group–treated with ursodeoxycholic acid; UDCA + LPS group–treated with ursodeoxycholic acid and endotoxin; CDCA group–treated with chenodeoxycholic acid; CDCA + LPS group–treated with chenodeoxycholic acid and endotoxin. One-way ANOVA, Bonferroni test, and Mann-Whitney test were performed. An asterisk (*) indicates significant differences compared to the LPS group ***p < 0.001, while the symbol (†) indicates significant differences compared to the control group †††*p* < 0.001.

The results showed that endotoxin significantly decreased (p < 0.001) AQP5 expression in rat lung tissue compared to the control group. Pretreatment with UDCA and CDCA led to a significant increase (p < 0.001) in the expression of this AQP in endotoxin-induced lung injury (Figures 14, 15A).

## 4 Discussion

The results of this study indicate that UDCA and CDCA exhibit antioxidative, anti-inflammatory, anti-apoptotic, and lung-protective effects in endotoxin-induced ALI. This is based on their ability to decrease prooxidative markers and increase antioxidative enzymatic activity in BALF; reduce the expression of NF-κB, caspase 3, and BAX; and increase the expression of BCL-2, AQP1, and AQP5 in lung tissue, thereby alleviating lung damage.

ROS play a significant role in the onset of inflammation and the progression of lung damage in cases of sepsis. Free radicals are produced due to the activity of pro-oxidative enzymes, lipid peroxidation, mitochondrial damage, hemodynamic changes, and protein kinase C activation (Abd Uljaleel and Hassan, 2023). A study by Johnson et al., using a rat model, demonstrated that intrapulmonary injection of ROS-generating enzymes results in ALI (Johnson et al., 1981). Excessive ROS production can damage cells in various ways. For example, ROS can damage lung epithelial cells by reacting with DNA, altering cell function and structure. Additionally, ROS can induce oxidative stress through the peroxidation of arachidonic acid present in cell membranes, thereby contributing to inflammation by inducing the production of inflammatory mediators (Bezerra et al., 2023). The analysis of pro- and antioxidant markers in BALF may be a more sensitive indicator of respiratory diseases compared to blood analysis (Fois et al., 2018). The 2017 study by Jarikre et al. evaluated oxidative stress parameters in BALF from healthy and pneumonic goats. Their results showed a significant increase in the levels of malondialdehyde (MDA), hydrogen peroxide (H_2_O_2_), and myeloperoxidase (MPO) in the supernatant of BALF pneumonic goats, along with a decrease in the levels of GSH and SOD activity (Jarikre et al., 2017). These results are in accordance with our findings in which endotoxin significantly decreased antioxidative and increased prooxidative markers in BALF. The reduction in nitrite levels in BALF in the LPS group could be influenced by several factors. The possible mechanism that could lead to the reduction of nitrite levels in BALF could be the increased degradation of nitrites. Specifically, during inflammation, reactive oxygen species (ROS) can rapidly react with nitrites, forming peroxynitrite (ONOO⁻) and other oxidative products (Robinson and Beckman, 2005). It could be also possible that nitrites were redistributed from BALF to other parts of the body, leading to lower levels in BALF.

There are reports of a wide range of protective effects of UDCA. It has been shown that this bile acid can modulate the immune response and epithelial ion transport (Mroz and Harvey, 2019). A study conducted by Niu et al. demonstrated that pretreatment with UDCA alleviated pathological and biochemical changes in ALI caused by fat embolism (Niu et al., 2020). The UDCA effectively inhibits pulmonary edema, inflammatory cell infiltration, production of pro-inflammatory cytokines, and oxidative stress (He et al., 2023). To investigate the effectiveness of CDCA in airway inflammation, a model of acute murine asthma was also used. The results of this study showed that the application of CDCA together with FXR (Farnesoid X receptor) in lung tissue reduces the severity of allergic airway disease in mice, as assessed by analyzing pathological and molecular markers associated with the disease (Shaik et al., 2015). The investigation of the effects of different bile acids on airway epithelial damage showed that cell viability depended on the concentration of bile acids, with a significantly higher concentration of CDCA required to induce cell death compared to lithocholic acid (LCA) (Aldhahrani et al., 2017). The results of our study showed that the application of UDCA and CDCA increases antioxidant protection (CAT, GSH), and reduces TBARS in BALF in endotoxin-induced ALI.

The results of the pathohistological analysis of the lungs showed significant lung tissue damage observed in the endotoxin-treated group. This is consistent with the study by Ali et al. (2020), who also showed a severe degree of lung damage in mice treated with endotoxin. Our results demonstrated that UDCA and CDCA protected lung cells from endotoxin-induced damage. This suggests the efficacy of pretreatment with UDCA and CDCA in reducing lung damage.

It has been shown that NF-κB activation is involved in the inflammatory response of the lung after exposure to endotoxin (Skerrett et al., 2004). Different studies have shown that UDCA has anti-inflammatory and immunomodulatory effects (Ainosah et al., 2020; Meng et al., 2018; Ko et al., 2017). A similar effect has been reported for CDCA (Dai et al., 2013). In competing with other bile acids for binding to G protein-coupled bile acid receptor 1 (GPCR-1, TGR5), UDCA successfully halted the activation of the NF-κB pathway leading to the regulation of inflammatory gene expression (Zhang et al., 2016). Studies have shown that UDCA reduces endotoxin-stimulated release of the pro-inflammatory cytokines and inhibits the expression of NF-κB in macrophages (Ko et al., 2017). Unlike UDCA, which consistently exhibited its cytoprotective effects, studies with CDCA have yielded varied results. Some studies have shown a proinflammatory role for CDCA (Gong et al., 2016; Horikawa et al., 2019). However, some studies with CDCA have demonstrated its cytoprotective activity. In our very recent study we demonstrated that UDCA and CDCA significantly reduced the production of pro-inflammatory cytokines, such as TNF-α, GM-CSF, IL-2, IFNγ, IL-6, and IL-1β, as well as the expression of nuclear factor κB (NF-κB) induced by LPS. Furthermore, treatment with these bile acids increased antioxidant activity (SOD, CAT, GSH) and decreased the concentrations of prooxidant markers H2O2 and O2– in plasma. Moreover, both bile acids alleviated LPS-induced liver injury. These findings confirmed the strong antioxidant role of UDCA and CDCA, which not only mitigate inflammatory processes but also prevent oxidative stress and tissue damage, including liver injury (Milivojac et al., 2024). The studies showed that CDCA exerts an inhibitory effect on the production of pro-inflammatory cytokines in endotoxin-stimulated macrophages via TGR5 receptor (Kawamata et al., 2003). Some studies demonstrated the anti-inflammatory effect of CDCA in tissues with the presence of FXR receptors, which are primarily activated by CDCA, suggesting that this effect is achieved through the activation of the anti-inflammatory properties of FXR itself (Mooranian et al., 2022). FXR is expressed in rat and human pulmonary endothelial cells, and it has been shown that in endotoxin-induced ALI, these receptors and their ligands (such as CDCA) play an important role in the repair of lung tissue following injury (Zhang et al., 2012). Our results showed that UDCA and CDCA can significantly reduce the excessive expression of NF-κB in lung tissue induced by endotoxin. Several studies complement our findings, highlighting the potential of bile acids, particularly UDCA, to modulate inflammation, oxidative stress, and apoptotic pathways. Studies have shown that UDCA improves histopathological changes associated with airway remodeling by modulating Th-2 cytokines, and by inhibiting apoptosis in airway epithelial cells (Işık et al., 2017). Another study demonstrated that UDCA treatment in ovalbumin-sensitized mice reduces eosinophilic airway inflammation through modulation of dendritic cells (DCs) and their interaction with T cells. UDCA enhanced interleukin-12 (IL-12) production in DCs and decreased cytokine production by T cells (Willart et al., 2012). The study by Moon and colleagues highlighted the broader protective effects of UDCA, where prior exposure to UDCA in patients with chronic liver disease reduced the risk of COVID-19 infection and severe outcomes. These findings suggest that UDCA’s anti-inflammatory properties extend beyond specific organ systems and may be linked to its regulatory effects on immune pathways (Moon et al., 2024). The cited studies complement our findings, underscoring the potential of bile acids, particularly UDCA, to modulate inflammation, oxidative stress, and apoptotic pathways.

Although there is a close relationship between the hydrophobicity and cytotoxicity of bile acids in the context of apoptosis induction, this correlation is not always consistent (Jang et al., 2022). Bile acids regulate the balance between death receptors and cytoprotective pathways, with the effect depending on the specific type of bile acid (Qiao et al., 2002). Studies have shown that UDCA affects the expression of more than 96 genes in rat hepatocytes, primarily those involved in cell cycle regulation and apoptosis (Castro et al., 2005). UDCA modulates the internal mitochondrial apoptosis pathway in various cells, as well as the external apoptosis pathway (Amaral et al., 2009). UDCA prevents BAX translocation, cytochrome c release, caspase activation, and the degradation of cellular components (Rodrigues et al., 1999). UDCA improves mitochondrial function by preserving their integrity, reducing the activity of caspases 9, 8, and 3, inhibiting ROS accumulation, and maintaining ATP levels, thereby significantly mitigating apoptosis (Huang, 2021). Additionally, UDCA reduces endoplasmic reticulum (ER) stress-induced apoptosis by decreasing Ca^++^ efflux and caspase-12 activation (Vang et al., 2014).

CDCA also plays an important role as a signaling molecule in various biological processes. This bile acid is more hydrophobic than UDCA, and since hydrophobic bile acids have been shown to induce apoptosis, a larger number of studies have suggested the pro-apoptotic effects of CDCA (Yerushalami et al., 2001; Shen et al., 2022). However, there are also studies indicating the anti-apoptotic effects of this bile acid. This suggests that non-toxic but hydrophobic bile acids do not induce apoptosis as they activate survival pathways, and NF-κB activation via the PI3 kinase-mediated pathway is crucial for inhibiting apoptosis. CDCA, at appropriate concentrations, increases mitochondrial membrane potential, prevents apoptosis, improves mitochondrial function, and reduces ROS and MDA levels (Xu et al., 2022). The results of our study showed that both bile acids exhibit anti-apoptotic effects by reducing the expression of pro-apoptotic markers caspase three and BAX and increasing the expression of the anti-apoptotic marker BCL-2 in endotoxin-induced ALI.

AQPs in the alveolar epithelium and endothelial cells of blood vessels facilitate the passage of fluids between the alveoli and their blood vessels (Verkman, 2007). The results of experiments in rats studying the role of AQP1 and AQP5 in endotoxin-induced ALI indicated reduced regulation of both AQPs, leading to increased inflammatory factors and apoptotic cells (Chen et al., 2016) Reduced regulation of AQP5 following primary endothelial injury of capillaries and alveolar epithelium leads to increased vascular permeability and cellular infiltration (Vassiliou et al., 2017). Modulation of AQPs under the influence of inflammatory mediators and inflammation signaling pathways involves transcriptional repression, covalent modifications, and changes in water channel transport. These mechanisms regulate inflammatory processes and diseases on an inflammatory basis (Rabolli et al., 2014). Reduced expression of AQP5 after LPS exposure in human bronchial epithelial cells is associated with the p38/JNK signaling pathway (Shen et al., 2012). The reduction in AQP5 expression after endotoxin treatment was also observed in submucosal gland cells of human airways (Shen et al., 2010). The pro-inflammatory cytokine TNF-α also showed a reduction in AQP5 expression in human bronchial epithelial cells (Mezzasoma et al., 2013)., and in cultured mouse lung epithelial cells, via tumor necrosis factor receptor 1 (TNFR1) activation and NF-κB translocation, along with interaction with binding sites on the AQP5 gene (Towne et al., 2001). AQP1 expression in rat lungs also decreases following endotoxin exposure. The reduction in AQP1 expression in endotoxin-induced RAW264.7 cells led to increased secretion of pro-inflammatory factors (Li et al., 2019).

The relationship between bile acids and AQPs is not fully understood, but some studies suggest that bile acids can affect AQP expression. AQPs are essential for bile formation because they facilitate water transport, and water makes up 95% of bile. In this context, the effects of bile acids on AQP in cholestasis have been investigated, although the function and regulation of AQPs in cholestasis remain unclear. Research has shown that endotoxin decreases AQP8 expression in rat hepatocytes, worsening cholestasis. Additionally, AQP8 and AQP9 expression is reduced in the livers of rats with ligated bile ducts, whereas increased AQP1 expression alleviates cholestasis. In patients with obstructive cholestasis, AQP10 expression is significantly reduced and negatively correlated with serum total bile acid levels, while increased AQP10 expression reduces liver damage. Conjugated bile acids (such as taurocholic acid) and inflammatory TNF-α decrease AQP10 expression, and NFκB p65/p50 directly binds to the AQP10 promoter, reducing its activity in the livers of patients with obstructive cholestasis (Liao et al., 2023).

The role and function of bile acids in AQP function and expression in endotoxin-induced ALI have not yet been documented. Our results showed a significant reduction in the expression of AQP1 and AQP5 in the lungs of endotoxin-treated rats. These findings suggest that their synthesis is impaired during ALI. In this study, endotoxin treatment exhibited significant apoptotic changes in rat lungs, and it appears that apoptosis of alveolar epithelial and endothelial cells affected the synthesis of these aquaporins. Therefore, decreased expression of AQP1 and AQP5 may lead to disruptions in water transport from pulmonary microvascular and alveolar epithelial cells, which could interfere with fluid movement in surrounding tissues and the clearance of alveolar fluid, resulting in exacerbated lung injury in endotoxin-induced ALI.

Our results showed that the administration of UDCA and CDCA increased the expression of AQP one and AQP5 in alveolar parenchyma in rat lungs, which may significantly contribute to mitigating endotoxin-induced lung injury. Examination of the effects of fasudil (a selective Rho kinase inhibitor) and Lipoxin A4 in endotoxin-induced ALI (Wang et al., 2019; Ba et al., 2019), and the effects of tanshinol (a hydrophilic polyphenol) on the lungs in a caecal ligation and puncture (CLP)-induced sepsis (Xu et al., 2018), have shown that they mitigate lung damage by increasing the expression of AQP5 by reducing the expression of NF-κB and the level of pro-inflammatory cytokines (TNF- α and IL-6). Considering the results of our recent research, which showed that UDCA and CDCA reduce the expression of NF-κB in the liver and the level of pro-inflammatory cytokines in the endotoxemia (Milivojac et al., 2024), NF-κB could be the main target of the beneficial effect of these bile acids on the expression of AQP1 and AQP5 in endotoxin-induced ALI. While these results suggest that pretreatment with UDCA and CDCA may protect the lungs from endotoxin-induced damage by regulating AQP1 and AQP5 expression, it is important to note the limitations of this study. The experimental design focused on elucidating mechanisms rather than exploring therapeutic actions. The bile acids were administered for several days before injury induction, which does not correspond to clinical scenarios where treatments are typically initiated after the onset of injury. This distinction underscores the need for further studies to evaluate the potential clinical applicability of these findings.

## 5 Conclusion

This experimental study reveals that pre-treatment with UDCA and CDCA exerts a beneficial effect in alleviating endotoxin-induced pulmonary damage in rats. Our findings suggest that UDCA and CDCA, despite being structurally distinct bile acids, may both contribute to the prevention of lung damage associated with endotoxemia. This hypothesis is supported by evidence showing that pre-treatment with UDCA and CDCA reduces oxidative stress and enhances the antioxidant enzyme capacity in BALF. Additionally, it decreases the expression of NF-κB, caspase 3, and BAX, while increasing the expression of BCL-2, AQP1, and AQP5 in lung tissue, and prevents lung damage.

## Data Availability

The raw data supporting the conclusions of this article will be made available by the authors, without undue reservation.
